# Supplementary material for: Magnetodynamics of short nanoparticle chains
Source: Sci Rep. 2025 Dec 9;15:43507. doi: 10.1038/s41598-025-22864-9 (PMC12695946; doi:10.1038/s41598-025-22864-9)
Supplement: Supplementary file 1 — Supplementary Information 1. [file 41598_2025_22864_MOESM1_ESM.docx]

**Magnetodynamics of few nanoparticle chains - Supplementary Materials (SM)**

# MPI imaging spatial resolution

The imaging spatial resolution is a function of the gradient field, *G*(*x,y,z*), and the point-spread-function, PSF^1,2^. The resolution advantage of SI over WI MNPs nanoparticles is presented in Fig. S1. The MPI images are acquired using a glass phantom (Fig. S1(a)) containing 10 *µL* colloidal MNP solution. 2D-slice images of the 3D glass phantom for the SI and WI MNPs are presented in Figs. S1(b) and S1(c), respectively. The four channels in the WI MNP image are blurred, but well resolved in the SI MNP image.

The point-spread-function (PSF), which correlates with the imaging spatial resolution, is measured in Fig. S1(d) and S1(e) for the WI and SI MNPs, respectively. SI MNPs exhibit exceedingly narrow PSFs at and above the threshold field up to ≈ 10*x* compared to the WI MNPs. Furthermore, SI MNPs display a greater dependency of the PSF width on magnetic field amplitude. Figure S1(f) show the fitted PSF full-width-half-maximum (FWHM) comparison for SI and WI MNPs. SI MNPs exhibit a sharp drop in the FWHM at the threshold field, which marks the onset of chaining behavior. Figure S1(g) is the ratio of the two curves in S1(f), or the resolution enhancement factor. This trend displays a strikingly similar behavior to that reported in previous work^6^, indicating a common origin in the particle response at variable field amplitude, despite being two independently synthesized colloidal systems using different synthesis protocols.


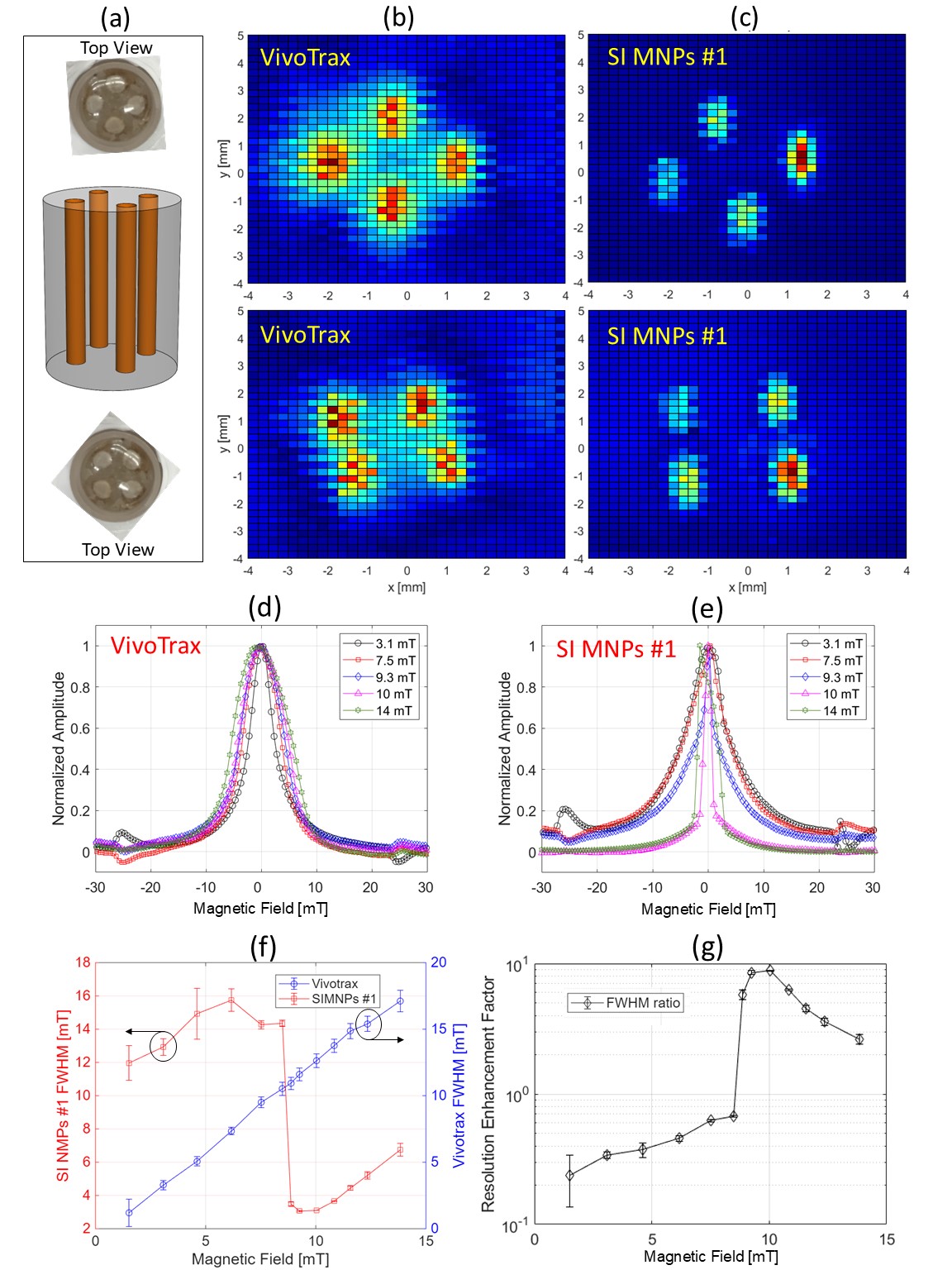


**Figure S1. (a)** A glass phantom for magnetic particle imaging (MPI) of colloidal MNP solution. Undiluted colloidal MNP samples fill the four wells in the glass phantom (gold color). **(b)** MPI image obtained using a commercial WI MNPs (VivoTrax) and **(c)** our SI MNPs #1 system. In the 2D-slice MPI image of the phantom, the four wells are blurred for the WI MNPs but clearly resolvable in the SI MNPs. The upper and lower rows of the MPI image in (b) and (c) correspond to rotated configurations of the phantom indicated by the top views of the phantoms in (a). Amplitude dependence of PSF for VivoTrax **(d)** and SI MNPs #1 **(e)** measured at 20 kHz. For the SI MNPs system, an abrupt change in the PSF at and above the threshold field is indicative of chaining. **(f)** The field dependence of the PSF’s fitted FWHM for SI MNPs #1 and WI MNPs (VivoTrax) samples. **(g)** The resolution enhancement factor by taking the ratio of the two curves in (f).

# SI MNP *m* vs *H* data

SI MNP #1 and SI MNP #2 were synthesized independently using two different methods (see Methods section for details), but displayed similar threshold behavior (Fig. S2(a)) and hysteresis (Fig. S2(b)) in the *m* vs *H* data.


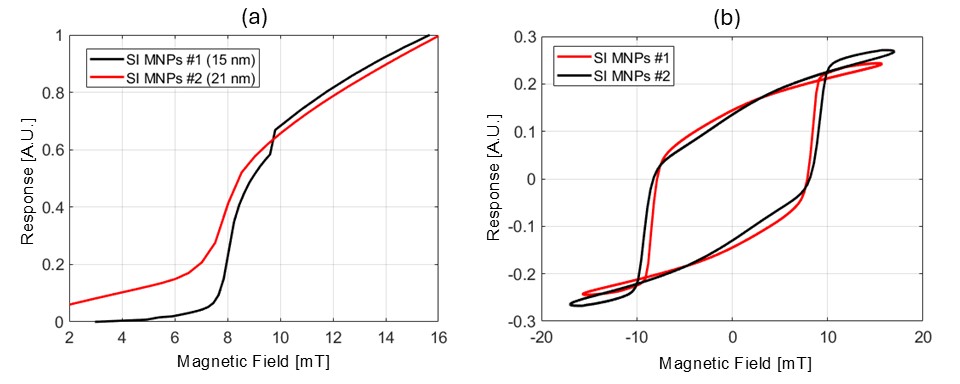


**Figure S2.** **(a)** Voltage response to indicate threshold behavior and **(b)** *m* vs *H* data for two independently synthesized SI MNP samples ("SI MNPs #1" and "SI MNPs #2"). The data are canted square loops displaying a positive linear slope and a square open loop.

# Micromagnetics simulations of switching times

The shape anisotropy of the chain produces an energy barrier to magnetization reversal: Figures. S3(a) and S3(b) studies the effect of this barrier on reversal. The green trace in Fig. S3(a) tracks the overall chain magnetization across a reversal event. The thicker purple trace, *H*_switch_, reports at each time index the strength of the field that would be necessary to complete the reversal from that magnetization configuration if the thermal field were removed. For example, in the initial configuration at t = 0 ns, a field of approximately 32 mT would be required. (Which is also the T = 0 K coercive field.) Not much changes for the first 20 ns, but between 20-25 ns *H*_switch_ drops, indicating that the magnetization configuration is effectively climbing up the energy landscape. At t = 22.5 ns the configuration clears the *µ*_0_*H*_app_ = 14 mT energy barrier. Beyond this point the reversal can proceed without the thermal field (Figs. 5(b) and 5(c)). Note that the shape of the energy landscape depends on the strength of the reversal field. To complete the reversal without either the applied or thermal fields requires waiting until t = 26 ns, where *M_x_/M_s_* = 0.

Figure S3(b) examines directly the energy terms in the simulation. The energy from the applied field (*E*_Zeeman_) and self-magnetostatic (*E*_demag_) terms are considered; the magnetization in each MNP is nearly uniform, so the exchange energy contribution is quite small. The shape anisotropy arises from the self-magnetostatic energy term (blue trace) and represents the barrier to reversal with no applied field. In this example that barrier is 120 zJ, or 30 *k_B_T* at 290 K. Applying a 14 mT reversing field reduces the barrier to approximately 12 *k_B_T*. This value can be combined with a *τ_N_* estimate from Table 5e and Eq. 4 to approximate *τ*_0_.


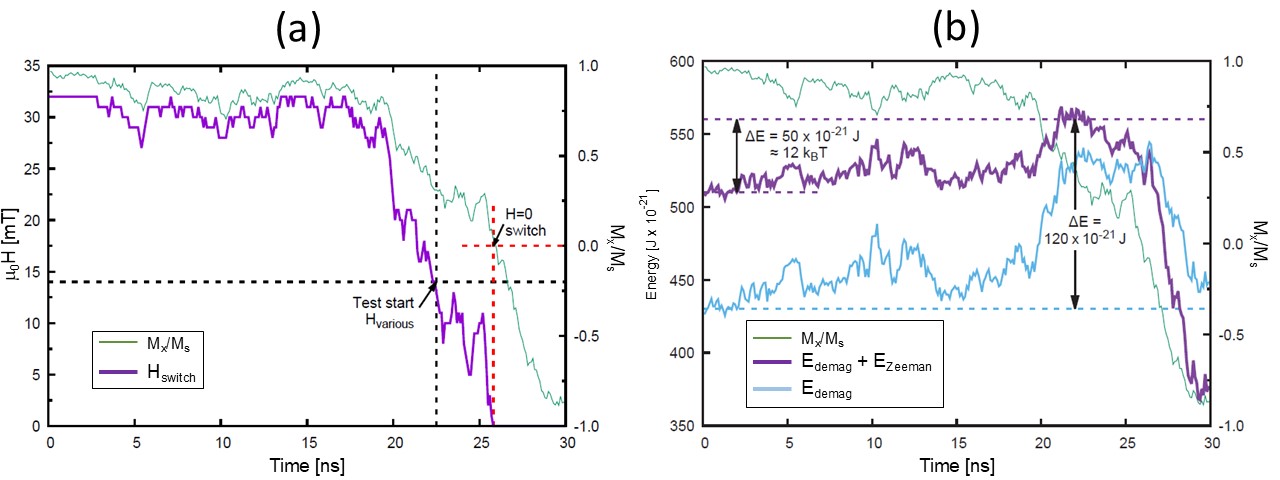


**Figure S3. (a)** Thermal reversal event used as base for subfigures 5(b) and 5(c) in the main text. The green trace corresponds to the solid purple trace in 5(b) and 5(c), but with the T = 290 K thermal field engaged throughout. The purple trace maps the minimal applied field needed to reverse the chain from that time index if the thermal field were deactivated. The point marked "Test start" is the starting point for subfigures 5(b) and 5(c). **(b)** The self-magnetostatic and applied field energy components for the reversal event depicted in subfigure (a).

# Concentration dilution effects for cryo-TEM experiments

To avoid complete attenuation of the electron beam in the cryo-TEM experiment, MNP sample concentrations were diluted by 10- to 100-fold from the native concentration. The SI MNPs behavior is preserved at these dilute concentrations (Fig. S4).


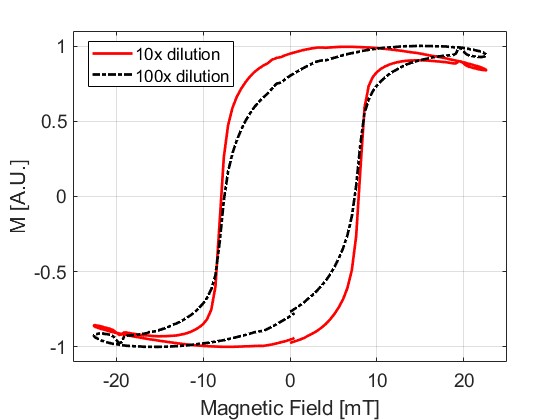


**Figure S4.** SI MNP #2 behavior is preserved at a 100-fold dilution for the cryo-TEM experiment. The canting (sloping) of *m* vs *H* data is removed by filtering out smaller particles that display only WI behavior.

# Determination of Particle Core and Hydrodynamic Diameter with TEM and DLS

Figure S5 shows the analysis of particle size distribution by TEM. The “SI MNPs #1” sample was imaged using a Tecnai T12 Spirit B12 electron microscope. The “SI MNPs #2” sample was imaged using a Phillips EM-400 electron microscope. Figure S5(a) shows a typical TEM image for SI MNPs #1. Line segments were drawn by hand in S5(b) using ImageJ across the apparent major axis of the nanoparticle core. The measurements were used to report the mean and standard deviation. A methodology using hand-drawn line segments was chosen over other algorithms (such as the “Analyze Particles” feature in ImageJ) because samples with strongly interacting magnetite cores were often observed to form aggregates. These appear as overlapping or obscured particles in TEM images. Hand-drawn line segments allowed discrimination between aggregates and single nanoparticle cores. Figure S5(c) shows the size distribution histogram and a log-normal fit to the data.

DLS was used to determine the hydrodynamic diameter of the particles. DLS samples were prepared by diluting the nanoparticles to an optically transparent level. Prior to DLS measurements, the strongly interacting/SFM samples were sonicated to break up potential aggregates in solution. DLS was performed after sonication. The measured hydrodynamic diameter, which is sensitive to the overall size of the magnetite core and surfactant coating, was larger than TEM measurements of the magnetite core alone. The relative standard deviation of size distributions from TEM and DLS was similar for each sample, though. The DLS measurements were performed with a Malvern Instruments Zetasizer Nano in the backscatter mode at 173° using a 633 nm wavelength laser.


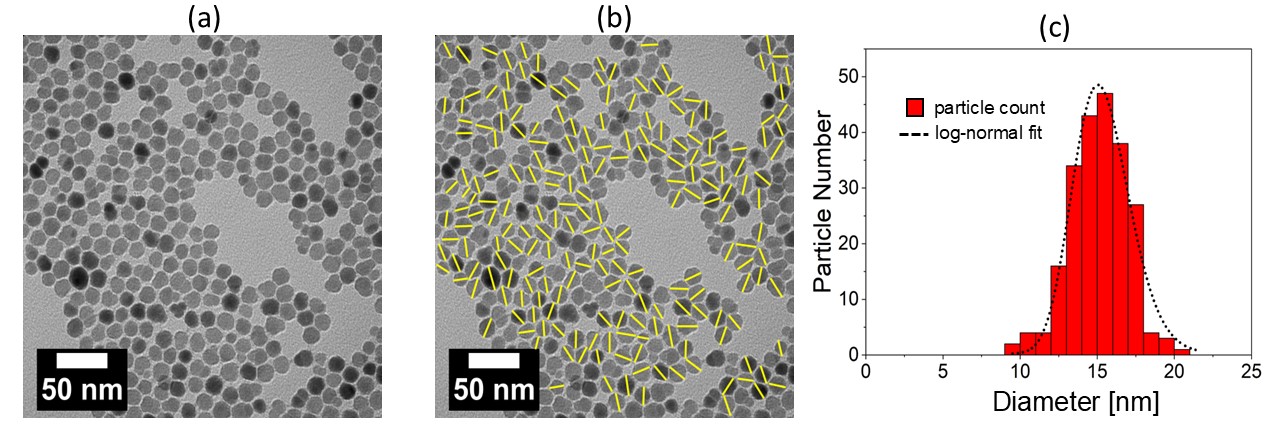


**Figure S5.** TEM analysis showing **(a)** the original TEM image, **(b)** the TEM image with hand-drawn ROIs, and **(c)** a histogram of sizes with a lognormal fit. Particles omitted from the ROI selection in (b) displayed overlap (or possible overlap) with other particle cores.

**References**

1. Goodwill, P. W. & Conolly, S. M. The x-space formulation of the magnetic particle imaging process: 1-d signal, resolution, bandwidth, snr, sar, and magnetostimulation. *IEEE Transactions on Med. Imaging* 29, 1851–1859, DOI:

10.1109/TMI.2010.2052284 (2010).

1. Croft, L. R., Goodwill, P. W. & Conolly, S. M. Relaxation in x-space magnetic particle imaging. *IEEE Trans Med Imaging* 31, 2335–2342 (2012).
2. Eberbeck, D., Wiekhorst, F., Steinhoff, U. & Trahms, L. Aggregation behaviour of magnetic nanoparticle suspensions investigated by magnetorelaxometry. *J. Physics: Condens. Matter* 18, S2829, DOI: 10.1088/0953-8984/18/38/S20 (2006).
3. Deissler, R. J., Wu, Y. & Martens, M. A. Dependence of brownian and néel relaxation times on magnetic field strength. *Med. Phys.* 41, 012301, DOI: <https://doi.org/10.1118/1.4837216> (2014).
4. Eggeman, A. S, Majetich, S. A. ,Farrell, D. & Pankhurst, Q. A. Size and Concentration Effects on High Frequency Hysteresis of Iron Oxide Nanoparticles. *IEEE Trans. Magn.* 43, 2451–2453, DOI: 10.1109/TMAG.2007.894127 (2007).
5. Tay, Z. W. *et al.* Superferromagnetic nanoparticles enable order-of-magnitude resolution & sensitivity gain in magnetic particle imaging. *Small Methods* 5, 2100796, DOI: 10.1002/smtd.202100796 (2021).
6. Abel, F. M. *et al.* Strongly interacting nano-ferrites for magnetic particle imaging and spatially resolved thermometry. *ACS Appl. Mater. & Interfaces* (Accepted, 2024).
